# Outcomes of Antiretroviral Therapy in Vietnam: Results from a National Evaluation

**DOI:** 10.1371/journal.pone.0055750

**Published:** 2013-02-15

**Authors:** Duc Bui Nguyen, Nhan Thi Do, Ray W. Shiraishi, Yen Ngoc Le, Quang Hong Tran, Hai Huu Nguyen, Nicholas Medland, Long Thanh Nguyen, Bruce Baird Struminger

**Affiliations:** 1 United States Centers for Disease Control and Prevention, Hanoi, Vietnam; 2 Vietnam Administration for HIV/AIDS Control, Ministry of Health, Hanoi, Vietnam; 3 Division of Global HIV/AIDS, Center for Global Health, Centers for Disease Control and Prevention, Atlanta, Georgia, United States of America; 4 Hanoi School of Public Health, Hanoi, Vietnam; British Columbia Centre for Excellence in HIV/AIDS, Canada

## Abstract

**Objectives:**

Vietnam has significantly scaled up its national antiretroviral therapy (ART) program since 2005. With the aim of improving Vietnam’s national ART program, we conducted an outcome evaluation of the first five years of the program in this concentrated HIV epidemic where the majority of persons enrolled in HIV care and treatment services are people who inject drugs (PWID). The results of this evaluation may have relevance for other national ART programs with significant PWID populations.

**Design:**

Retrospective cohort analysis of patients at 30 clinics randomly selected with probability proportional to size among 120 clinics with at least 50 patients on ART.

**Methods:**

Charts of patients whose ART initiation was at least 6 months prior to the study date were abstracted. Depending on clinic size, either all charts or a random sample of 300 charts were selected. Analyses were limited to treatment-naïve patients. Multiple imputations were used for missing data.

**Results:**

Of 7,587 patient charts sampled, 6,875 were those of treatment-naïve patients (74.4% male, 95% confidence interval [CI]: 72.4–76.5, median age 30, interquartile range [IQR]: 26–34, 62.0% reported a history of intravenous drug use, CI: 58.6–65.3). Median baseline CD4 cell count was 78 cells/mm^3^ (IQR: 30–162) and 30.4% (CI: 25.8–35.1) of patients were at WHO stage IV. The majority of patients started d4T/3TC/NVP (74.3%) or d4T/3TC/EFV (18.6%). Retention rates after 6, 12, 24, and 36 months were 88.4% (CI: 86.8–89.9), 84.0% (CI: 81.8–86.0), 78.8% (CI: 75.7–81.6), and 74.6% (CI: 69.6–79.0). Median CD4 cell count gains after 6, 12, 24, and 36 months were 94 (IQR: 45–153), 142 (IQR: 78–217), 213 (IQR: 120–329), and 254 (IQR: 135–391) cells/mm^3^. Patients who were PWID showed significantly poorer retention.

**Conclusions:**

The study showed good retention and immunological response to ART among a predominantly PWID group of patients despite advanced HIV infections at baseline.

## Introduction

Vietnam has a population of more than 84 million and an estimated 2011 adult (15–49 years) HIV prevalence of 0.45% [1]. However, the epidemic is primarily concentrated in most-at-risk populations, including people who inject drugs (PWID), commercial sex workers (CSW), and men who have sex with men (MSM). There were an estimated 248,500 people living with HIV/AIDS in Vietnam in 2011. Among PWID the HIV prevalence was 25.5%; among CSW, 6.8%; and among high-risk MSM, 11.2% [2]. National size estimates of those high-risk populations were as high as 336,000 for PWID, 101,000 for CSW, and 393,000 for MSM [2].

In 2005, in response to the epidemic, the Ministry of Health (MOH) established the Vietnam Administration for HIV/AIDS Control (VAAC) to coordinate HIV response activities. The MOH developed guidelines for diagnosis and treatment of HIV/AIDS and established outpatient clinics (OPC) where HIV-infected patients have been offered free care and treatment services.

Nationwide scale-up of a free antiretroviral therapy (ART) program began in 2005 through a network of HIV outpatient clinics (OPCs) using a CD4 T-cell count threshold for ART initiation of 200 cells/mm^3^. As of December 2011, 57,600 patients were receiving ART in 305 adult OPCs. This accounted for 53% of those estimated to be in need of treatment [1]. ART services have been funded by three major sources: (1) the U.S. President’s Emergency Plan for AIDS Relief (PEPFAR), (2) the Global Fund for AIDS, Tuberculosis and Malaria (GF), and (3) the National Target Program for HIV/AIDS Prevention and Control (NP).

With the national ART program still in the process of scale-up, it is important to evaluate the ART program outcomes so that program weaknesses can be addressed. The goal of this evaluation was to assess the clinical and programmatic outcomes of adult HIV-infected patients after ART initiation and factors associated with those outcomes.

## Methods

### Vietnam ART Program and Guidelines

The current 2009 Vietnam MOH’s *Guidelines for Diagnosis and Treatment of HIV/AIDS* and *National ART Protocol* state that all HIV-infected persons who are at least 16 years of age are eligible for care and treatment services at adult OPCs. Patients are referred from voluntary counseling and testing sites, hospitals, general medicine clinics, or peer outreach groups. Once registered for HIV care services, patients are followed every three to six months with clinical exams and laboratory testing until eligible for treatment. In 2009, criteria for starting ART were WHO stage I or II with CD4 cell count less than 250 cells/mm^3^, WHO stage III with CD4 cell count less than 350 cells/mm^3^, or WHO stage IV regardless of CD4 cell count [3]. The CD4 threshold for starting ART among patients with WHO stage I or II was increased to 350 cells/mm^3^ in 2011. According to the national guidelines in 2009, MOH-preferred first-line regimens included combinations of zidovudine (AZT) or stavudine (d4T), lamivudine (3TC), and nevirapine (NVP) or efavirenz (EFV). However, stavudine was phased out in 2011 and the combination of tenofovir, lamivudine, and efavirenz was recommended as the preferred first line regimen.

OPCs are located at three levels of the health care system: central, provincial and district levels. A single treatment protocol is applied at all levels. All clinical data are recorded in the MOH’s standardized paper-based forms and patient charts. Before ART initiation, all patients undergo a medical examination, which includes complete medical history, physical examination, chest radiograph, complete blood count (CBC), liver transaminases, hepatitis B surface antigen and hepatitis C antibody testing. CD4 cell count testing is available to patients in most clinics, but HIV viral load testing is not routinely available. Adherence counseling sessions are provided to familiarize patients with regimens and adverse events and to promote adherence. After starting ART, patients are required to return weekly during the first four weeks to pick-up their medications, then every two weeks for the next month, and monthly thereafter. At each visit weight, WHO stage, and clinical signs and symptoms are recorded. Routine testing for CBC, liver transaminases and CD4 cell count is performed at 6-month intervals from ART initiation or before switching to second-line therapy. Co-trimoxazole prophylaxis is recommended to patients in WHO stage III or IV regardless of CD4 cell count or patients in WHO stage I or II with CD4 cell count less than 200 cells/mm^3^. During the clinical course of care and treatment, patients are generally offered a variety of HIV services, including prevention of mother-to-child transmission, TB/HIV screening and treatment, peer outreach, and needle and syringe programs.

### Services for PWID

Because the majority of HIV infections occur among PWID, services for PWID are a priority of the VAAC. Needle and syringe programs are available in 35 of 64 provinces. Methadone maintenance therapy (MMT), which started in 2008 in 2 pilot provinces, expanded to 41 clinics in 11 provinces by the end of 2011 with more than 7,000 PWID receiving MMT treatment. However, since the MMT program was in pilot status at the time of this study, data on ART patients on MMT were limited. Other programs such as the 100% Condom Use Program, voluntary counseling and testing, and outreach programs are also generally available.

### Design

This study was a retrospective cohort analysis of adult patients who started ART at OPCs in Vietnam. Data were abstracted from patient charts.

### Patient Eligibility

All adults 16 years of age or older at the time of ART initiation who started ART at least 6 months prior to the date of chart abstraction were eligible for inclusion in the evaluation. Analyses were limited to ART-naïve patients, defined as patients who reported no antiretroviral use before ART initiation at participating clinics.

### Sample Size

A sample size of 1,537 was calculated as needed to estimate the proportion of adults who are alive and on therapy 12 months after ART initiation, expecting a proportion of approximately 80%, using a finite population of 28,000, which was the total number of patients on ART in the country at the time of the study protocol development, so that a 95% confidence interval equal to the sample proportion ±2% is produced.

### Site and Sample Selection

All OPCs providing ART for at least 1 year and with 50 or more adults on ART as of March 2009 were eligible for inclusion in the sampling frame. Of the 173 adult ART clinics active in Vietnam at the time, 120 (69%) met the eligibility criteria. These 120 clinics included 39 supported by the U.S. President’s Emergency Plan for AIDS Relief (PEPFAR) (33%), 50 supported by the Global Fund (GF) (42%), and 31 by the National Program (NP) (25%) providing ART to a total of 25,000 patients (89% of all ART patients countrywide). The 39 PEPFAR-funded clinics accounted for 15,500 (62%) of the patients, while GF-funded clinics accounted for 6,000 (24%) patients. Due to funding and staff-time limitations, we randomly selected 30 (25%) of the 120 eligible OPCs. A multi-stage sampling strategy was applied: In the first stage, we randomly selected 12 of 39 PEPFAR-funded clinics, 10 of 50 GF-funded clinics and 8 of 31 NP-funded clinics using probability proportional to size (PPS). We sampled clinics according to funding partner because in the early phase of the national ART program there were differences between partners regarding clinic location and types (e.g., clinics supported by PEPFAR were usually at the provincial level or in large cities), and level of technical support (clinical and lab support were generally more available to PEPFAR and GF supported clinics); however, in recent years those differences have become less significant as a result of increased coordination of services by VAAC. This approach helped improve the national estimates and allowed for partner-specific estimates which were useful for feedback related to partner-specific program implementation. However, it was not our intention to compare the differences between partners. In the second stage of the evaluation, for clinics with more than 300 eligible ART patients, we randomly selected a sample of 300 patients from a clinic registry of all patients who ever started ART, and for clinics with fewer than 300 eligible ART patients we selected all patients from that clinic.

### Data Collection

Chart abstractions were conducted at the 30 selected clinics. Re-abstraction and validation was performed on 10% of the randomly re-selected charts. No personal identifying information was collected. The data were anonymous and unlinked. All data were double-entered into an Epi-Info database (Epi Info v.3.3.2 for Windows). SAS 9.2 (SAS Institute Inc., Cary, NC) was used for comparison and resolution of double-entered data.

### Ethical Review

Due to the nature of the evaluation, a waiver of informed consent for medical record abstraction was requested. The survey instrument, including the waiver of informed consent, was approved by the Vietnam Ministry of Health and the Institutional Review Board of U.S. Centers for Disease Control and Prevention (Atlanta, Georgia).

### Statistical Analysis

All analyses were performed using SAS 9.2 (SAS Institute Inc., Cary, NC) and Stata/IC 10.1 (StataCorp, 2009, Stata Statistical Software: Release 10.1, College Station, TX). Statistical analyses were weighted and accounted for the complex design of the evaluation. Domain analyses were performed to appropriately account for the analysis of subpopulations.

Missing data were assumed *missing at random* (MAR) [4] and were imputed multiply via chained equations using the ice [5], [6], [7] procedure in Stata. Twenty imputed datasets were created and estimates were combined according to Rubin’s rules [4] using the mim procedure [8]. The imputation model included the Nelson-Aelen estimate of cumulative hazard [9], baseline demographic and clinical variables, and the event indicator. Time-to-event data were complete for all individuals. Twenty-five patients were lost to follow-up (LTFU) after their first clinic visit and were assigned one day of person-time. Stratified Kaplan-Meier curves were used to graphically assess retention proportions. These analyses were limited to the first imputed dataset. Cox proportional hazards models were used to identify factors (year of ART initiation, sex, intravenous drug use, baseline CD4 cell count, baseline WHO stage, baseline body mass index (BMI), treatment for active tuberculosis disease (TB), baseline hemoglobin, initial ART regimen, clinic type, clinic location, clinic size) associated with attrition. *Attrition* was defined as discontinuation of ART due to death, loss to follow-up, or discontinuation of ART medications while remaining in care. Hazard ratios (HRs), 95% confidence intervals (CI) and p-values were calculated. Deaths were recorded if documented in the patient record. Patients who had not presented to the clinic for more than 90 days since their last recorded visit were considered lost to follow up. For this study, *retention* referred to patients known to be alive and receiving highly active ART at the end of the follow-up period. For time-to-event analyses, patients who transferred to another clinic were censored at the date of transfer. However, for estimating retention proportions at 6, 12, 24, 36, and 48 months, transfers were excluded from the retention analysis. Baseline CD4 cell count was defined as the value on the date closest to ART start date, but not more than 182 days prior to that date or more than 1 day after that date. For clinical stages, weight and CD4 cell count at 6 months, 12 months, and 24 months of follow-up were selected; when multiple values were available the values obtained on the dates closest to 182 days, 365 days, and 730 days were selected.

## Results

### Characteristics at Clinic Registration

From March 2010 to May 2010, data from records of 7,587 eligible patients at 30 clinics were abstracted; preliminary results of the full cohort were presented elsewhere [10]. However, we focused the analyses on treatment-naïve patients (N = 6,875). All patients, except one, received ART between 2005 and 2009. At registration, the majority of patients were in WHO stage III or IV (69.7%, 95% CI: 64.4–75.1) and had a median CD4 cell count of 73 cells/mm^3^ (IQR: 28–128). Median CD4 cell count at registration for patients who started ART was 39 cells/mm^3^ in 2005, 51 cells/mm^3^ in 2006, 66 cells/mm^3^ in 2007, 75 cells/mm^3^ in 2008, and 87 cells/mm^3^ in 2009. Median waiting time from registration to start of ART was 60 days (IQR: 28–149) with a reduction from 137 days in 2005 to 56 days in 2009.

### Characteristics at ART Initiation

Table S1 illustrates the clinical and laboratory characteristics of patients at ART initiation. Both original and imputed results are presented. The following text reports weighted imputed data.

The majority of patients were male (74.4%, 95% CI: 72.4–76.5), median age was 30 years (IQR: 26–34), and 62.0% (95% CI: 58.6–65.3) reported a history of intravenous drug use (IDU).

Active TB was relatively common at ART initiation (13.9%, 95% CI: 10.4–17.3). Mean BMI was 18.5 (95% CI: 17.1–20.1).

Most patients started ART in the setting of late presentation of HIV infection. WHO stage III and IV was recorded in 46.9% (95% CI: 42.6–51.1) and 30.4% (95% CI: 25.8–35.1) of patients, respectively, and median CD4 cell count was 78 cells/mm^3^ (IQR: 30–162). Only 15.9% (95% CI: 13.3–18.5) of patients had CD4 cell count ≥200 cells/mm^3^ at ART initiation.

Median hemoglobin was 121 g/l (IQR: 105–136) and was lower among women than men. Grade 4 anemia (hemoglobin <65 g/l) was seen in 1.9% (95% CI: 1.1–2.6) of patients.

Prevalence of hepatitis B infection was 14.2% (95% CI: 11.9–16.4); prevalence of hepatitis C infection was 39.6% (95% CI: 34.8–44.3). However, data on hepatitis testing was missing for many patients (51.4% for hepatitis B and 65.4% for hepatitis C). Most patients started d4T/3TC/NVP (74.3%, 95%CI: 68.9–79.7) or d4T/3TC/EFV (18.6%, 95%CI: 14.5–22.7).

Table S1 illustrates the baseline characteristics between patients who reported injecting drug use (IDU) and patients who did not report IDU. PWID tended to present with lower CD4 cell count (69 vs. 96 cells/mm^3^), more severe WHO stages (82.7% vs. 68.4% in WHO stage III/IV), higher prevalence of TB (16.2% vs. 10.0%), and HCV infection (52.6% vs. 18.1%).

### Retention

Table S2 describes death, lost-to-follow-up, transfer out and retention rates of patients after 6, 12, 24, 36 and 48 months. Retention at 6 and 12 months was high for all patients (88.4% and 84.0%, respectively) as well as for PWID (86.7% and 81.5%) and patients who did not report IDU (92.6% and 90.1%). However, by 24, 36 and 48 month, retention among non-IDU was significantly better.

Factors associated with attrition are described in Table S3 with crude and adjusted hazard ratios (AHR) and 95%CI. Male sex (AHR 1.59, 95%CI: 1.26–1.99), history of IDU (AHR 1.58, 95%CI: 1.25–2.01), late presentation at WHO stage IV (AHR 1.48, 95%CI: 1.22–1.81), lower CD4 cell count (AHR 1.61, 95%CI: 1.28–1.93 for CD4 cell count between 50 and 200 cells/mm^3^; AHR 2.40, 95%CI: 1.90–3.04 for CD4 below 50 cells/mm^3^) and lower BMI (AHR 1.75, 95%CI: 1.51–2.03) were associated with attrition in the adjusted model. Having active TB at ART initiation and having lower hemoglobin were associated with attrition in the unadjusted analysis, not in the adjusted hazard model. Similar factors (male, low CD4 cell count, late stage, low BMI) were found associated with attrition for the PWID sub-population. Site factors, including clinic type (provincial vs. district), clinic location (rural vs. urban), and clinic size (i.e., number of patients) were also tested, and none of them showed significant association with attrition. However, data related to the MMT program was not available for analysis.

A Kaplan-Meier plot constructed to compare retention rates between IDUs and non-IDUs (Figure S1) showed that patients with a history of IDU had lower retention rates than patients without it.

### Immune Reconstitution Under ART

Median CD4 cell count gains were 94 cells/mm^3^ after 6 months, 142 cells/mm^3^ after 12 months, 213 cells/mm^3^ after 24 months, 254 cells/mm^3^ after 36 months, and 264 cells/mm^3^ after 48 months of ART (Table S2). The gain was rapid in the first 24 months and slower in the 3^rd^ year of ART. Patients of both sexes and patients with or without a history of IDU experienced increases in CD4 cell count. However, patients with an IDU history generally had poorer immune reconstitution than patients without a history of IDU.

## Discussion

We described cohorts of Vietnamese HIV-infected patients with late presentation to care. Most patients registered at OPCs already with advanced stages of HIV infection. These findings were similar to reports from other resource-limited settings, including sub-Saharan Africa [11], [12], [13], [14], [15], [16], [17], [18], China [19], India [20], Thailand [21] and Haiti [22]. Even after several years of experience managing the national ART program, late presentation remains problematic in Vietnam as it does in many other countries [12], [20]. Although Vietnam’s ART program scaled up rapidly from 2005 to 2009, the median CD4 cell count at clinic registration and before ART remained under 100 cells/mm^3^ throughout this period. Significant stigma and discrimination towards HIV-infected persons, weak Information/Education/Communication programs and poor linkage between counseling and testing services and OPCs may be factors contributing to late access to care. Moreover, the scale-up rate in the early stage of the ART program (i.e., 2004–2006) was slow due to time and resources needed to set up OPCs, train health care workers, and to gain experience for more rapid ART initiation and wider coverage.

Although patients in this cohort presented with advanced infections, 12-month retention on treatment was high (>80%). Such retention is similar to those reported from a systematic review of cohorts in sub-Saharan Africa [23], as well as country specific reports from Rwanda [14] and South Africa, Malawi and Côte d’Ivoire [15]. The national ARV program in Thailand also achieved a high survival rate of 0.89 after 1 year and 0.78 after 5 years [21]. Moreover, despite low CD4 cell counts at baseline, our evaluation showed a positive immunologic response to ART. The gain in CD4 cell count continued steadily through the course of ART, with a median gain of 94 cells/mm^3^ at six months and 142 cells/mm^3^ at 12 months. The trend continued at 24, 36, and 48 months. This promising response was similar to results found in sub-Saharan Africa [11], [12], [14], [17], China [19], Thailand [21], and Haiti [22].

This programmatic evaluation also identified factors associated with attrition. Being male, having a history of IDU, low CD4 cell count, high WHO stage, and low BMI were risk factors for attrition. Some of those, such as being male, low CD4 cell count, high WHO stage, and low BMI were common risk factors reported in resource-limited settings [11], [14], [17]. History of IDU was a strong risk factor for attrition, which could be explained by poor adherence, active drug use, or co-morbidities. Clinic factors (i.e., size, type, location) were not found to be associated with attrition, although factors specific to PWID (e.g., access to MMT) were not available for analysis because the MMT program was still being piloted at the time of the study.

The important difference between this study and other ART cohorts is the high proportion of PWID. IDU has been the major route of transmission in Vietnam [24]. PWID in this evaluation presented with more advanced HIV infections and were more likely to have evidence of HCV co-infection. Cohorts of PWID in other countries have reported similar findings: PWID were more likely to have lower nadir CD4 cell count measurements, present with AIDS defining illnesses [25], [26], [27], have higher rates of active TB [28], have higher prevalence of hepatitis/HIV co-infections [29], [30], and receive HIV diagnosis late [31]. In Vietnam, major barriers for PWID to access care and treatment include poor socioeconomic status, discrimination (and in some cases, self-discrimination), lack of knowledge about HIV disease and benefits of early testing and care, concern about confidentiality, lack of transportation, limited availability of IDU-centered services such as MMT and outreach support (Family Health International 360, unpublished data).

Although retention of PWID at 12 months was also promising (81.5%), fewer PWID than non-PWID were retained over the course of ART: while non-PWID maintained retention rates of 88.2% at 3 years and 85.7% at 5 years, the retention rates of persons with a history of IDU were significantly lower, only 67.4% at three years and 63.3% at five years. Additionally, PWID were found to have less robust immune reconstitution than patients without a history of IDU (Table S2). Other reports have shown that persons with a history of IDUs were less likely to achieve viral load suppression [26], more likely to have treatment interruption, [32] and lower CD4 gain [33]. In our evaluation, contributing factors to less favorable outcomes of PWID might include ongoing drug use, co-morbidities or poor adherence, although data were not available to identify any associations. Because adherence data were inconsistently recorded in the charts, we were not able to analyze adherence among PWID. Anecdotal reports from clinics participating in the evaluation showed that ongoing drug use accounted for a number of patients who had died of presumed overdose or had been confined to drug treatment centers without continuing access to comprehensive care and ART. Efforts have been made by VAAC to provide care to drug treatment center residents and maintain ART access. Expansion of MMT and other harm reduction programs targeting PWID with better linkages to OPCs is underway and should help increase access to care and improve adherence among PWID on ART.

The present evaluation had several limitations. First, data quality was suboptimal because the source was patient charts used by health care providers. Indeed, many data were missing, including baseline CD4 cell count, weight, WHO stage, and viral hepatitis sero-status. Testing was not always routinely performed and therefore results were not always recorded. CD4 measurement and viral hepatitis testing was not widely available at all clinics, especially early in the ART program (i.e., 2004–2006). Another important data quality issue was related to the adherence of healthcare workers to national guidelines. Although Vietnamese national guidelines recommend WHO stage, weight, clinical status, and adverse events to be recorded at every patient visit, we found that a considerable number of charts were missing this data (Table S1). ART laboratory monitoring was also often not performed per guidelines, similar to another study [34]. Critical tests such as CD4 cell count, liver transaminases, and hemoglobin sometimes were not done at 6-month intervals as recommended. Some patients did not have any CD4 measurements at follow up or had CD4 measurements done more than 1 year after ART initiation. These quality issues were reported back to VAAC to help establish a more rigorous quality control program with the goal of improving quality of care.

A second limitation was that we were not able to assess what happened to patients who were lost to follow-up or to assess the reasons for deaths as the charts did not routinely record these data. Other studies tracking loss to follow-up reported that one third [35], [36] to half [37] of LTFU patients died and many of the patients were untraceable. A meta-analysis of studies from Africa, Asia and Latin America reported 40% of LTFU patients had died, and among patients for whom re-contact was successful, reasons for not returning to clinics included transfer to other programs, financial difficulties, improving or decreasing health status and stigma [38]. In the context of Vietnam, we would expect similar circumstances. Many of the LTFU patients may have already died. Especially in this population of PWID, active drug use may contribute to loss to follow up, either because patients died or were admitted to government drug treatment centers.

The third limitation was the ascertainment of IDU history. Many patients who had used intravenous drugs may not have revealed their history, leading to a falsely low proportion of PWID. High HCV co-infection among reported non-PWID (18.1%) may serve as a proxy for IDU status. Also, we were also not able to obtain data on whether patients continued intravenous drug use while on ART or whether they had access to MMT.

Despite these limitations, the evaluation provided significant insights and useful lessons for the Vietnamese ART program. The evaluation served as an assessment of data quality at OPCs and based on its results recommendations were made to VAAC to 1) revise the current patient chart format to be more user-friendly and to capture additional important patient monitoring indicators; 2) provide training to OPC staff to increase data use for internal quality improvement; 3) strengthen patient monitoring; 4) emphasize the importance of routine quality control activities; and 5) consider an evaluation of LTFU patients to better understand their outcomes and reasons for LTFU. Although the evaluation did not provide data to assess specific factors associated with less desirable outcomes among PWID, such as ongoing drug use, lack of access to MMT, and adherence, we believe that strengthening IDU-specific interventions, such as linkage to MMT which is becoming rapidly more available, as well as counseling and treatment support, will help reduce LTFU and improve treatment outcomes. In an effort to increase early access to ART, VAAC revised the national guidelines in 2011 to raise the threshold of CD4 cell count for ART initiation to 350 cells/mm^3^, and it recommended ART to all patients with active TB regardless of the counts. The Vietnam MOH also committed to piloting the WHO/UNAIDS “Treatment 2.0″ initiative [1]. Up to now, the MMT program has been expanded to 41 clinics with stronger linkages to OPCs. As a result of the combination of these efforts, PWID may expect to experience more favorable outcomes.

### Conclusion

Data from this routine program evaluation of ART scale-up in Vietnam demonstrated a high retention rate and good immune reconstitution in response to treatment, indicating a high level of program quality in this predominately PWID population. However, efforts to promote earlier identification of HIV-infected persons and earlier initiation of ART remain a priority, as well as efforts to improve the quality of clinic services and data to further reduce LTFU among PWID.

## Supporting Information

Figure S1 **Retention of patients with and without history of IDU.** This Kaplan-Meier plot compared retention rates between IDUs and non-IDUs. The figure showed that patients with IDU history had lower retention rates than patients without IDU history. Analysis was performed on imputed dataset 1. Abbreviation: IDU, intravenous drug use.
(TIF)

Table S1 **Characteristics of patients before ART initiation.** (1) Unweighted. Abbreviation: IDU, intravenous drug use; ART, antiretroviral therapy; CI, confidence interval; IQR, interquartile range; TB, active tuberculosis; WHO, World Health Organization; BMI, body mass index.
(DOCX)

Table S2 **Patient outcomes on ART: retention and immunologic response.** Note: These analyses are not based on imputed data. (1) Percentages, 95% confidence interval presented in parentheses. (2) Medians, IQR presented in parentheses. Abbreviation: IDU, intravenous drug use; ART, antiretroviral therapy; CI, confidence interval; IQR, interquartile range; LTFU, loss-to-follow up.
(DOCX)

Table S3 **Factors associated with attrition.** (1) average sample size across 20 imputed datasets. Abbreviation: PY, person-years; HR, hazard ratio; AHR, adjusted hazard ratio; IDU, intravenous drug use; ART, antiretroviral therapy; CI, confidence interval; IQR, interquartile range; TB, active tuberculosis; WHO, World Health Organization; BMI, body mass index.
(DOCX)
