# Supplementary figures and images for: Outcomes of Antiretroviral Therapy in Vietnam: Results from a National Evaluation
Source: PLoS One. 2013 Feb 15;8(2):e55750. doi: 10.1371/journal.pone.0055750 (PMC3574016; doi:10.1371/journal.pone.0055750)

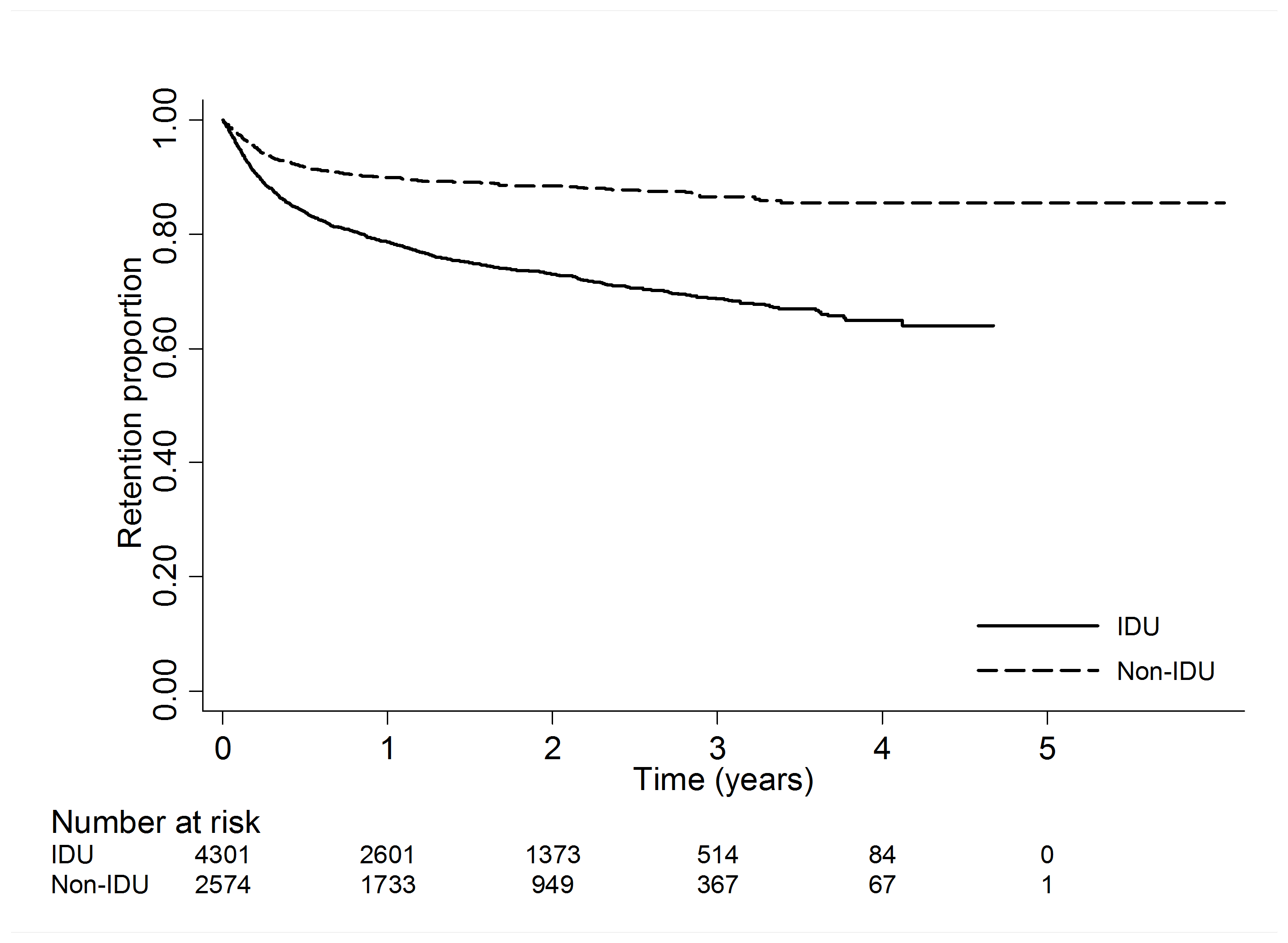

Supplement: Figure S1 — Retention of patients with and without history of IDU. This Kaplan-Meier plot compared retention rates between IDUs and non-IDUs. The figure showed that patients with IDU history had lower retention rates than patients without IDU history. Analysis was performed on imputed dataset 1. Abbreviation: IDU, intravenous drug use. (TIF) [file pone.0055750.s001.tif]
