# Supplementary material for: Outcomes of Antiretroviral Therapy in Vietnam: Results from a National Evaluation
Source: PLoS One. 2013 Feb 15;8(2):e55750. doi: 10.1371/journal.pone.0055750 (PMC3574016; doi:10.1371/journal.pone.0055750)
Supplement: Table S1 — Characteristics of patients before ART initiation. (1) Unweighted. Abbreviation: IDU, intravenous drug use; ART, antiretroviral therapy; CI, confidence interval; IQR, interquartile range; TB, active tuberculosis; WHO, World Health Organization; BMI, body mass index. (DOCX) [file pone.0055750.s002.docx]

Table S1. Characteristics of patients before ART initiation

|  |  |  |  | Multiple Imputation (N = 6,875) | | |
| --- | --- | --- | --- | --- | --- | --- |
|  |  |  | All | All | IDU | Non-IDU |
|  | n | N | Percentage (95% CI) or Median (IQR) | Percentage (95% CI) or Median (IQR) | Percentage (95% CI) or Median (IQR) | Percentage (95% CI) or Median (IQR) |
| Gender |  |  |  |  |  |  |
| Female | 1,785 | 6,871 | 25.5% (23.5-27.6) | 25.6% (23.5-27.6) | 6.5% (5.3-7.8) | 56.9% (53.0-60.7) |
| Male | 5,086 | 6,871 | 74.5% (72.4-76.5) | 74.4% (72.4-76.5) | 93.5% (92.2-94.7) | 43.1% (39.3-47.0) |
| Missing^1^ | 4 | 6,875 | 0.07% | -- | -- | -- |
| Age at ART start (years) |  |  |  |  |  |  |
| All | 6,875 | 6,875 | 30 (26-34) | 30 (26-34) | 29 (26-33) | 30 (27-35) |
| Missing^1^ | 0 | 6,875 | 0.0% | -- | -- | -- |
| IDU |  |  |  |  |  |  |
| Yes | 2,521 | 4,139 | 61.3% (57.0-65.6) | 62.0% (58.6-65.3) | -- | -- |
| Missing^1^ | 2,736 | 6,875 | 39.8% | -- | -- | -- |
| Treatment for active TB |  |  |  |  |  |  |
| Yes | 820 | 6,875 | 13.9% (10.4-17.3) | 13.9% (10.4-17.3) | 16.2% (12.4-20.0) | 10.0% (6.8-13.3) |
| Missing^1^ | 0 | 6,875 | 0.0% | -- | -- | -- |
| WHO stage |  |  |  |  |  |  |
| I/II | 1,470 | 6,515 | 22.8% (18.6-27.1) | 22.7% (18.5-26.8) | 18.4% (14.5-22.4) | 33.6% (27.7-39.5) |
| III | 3,075 | 6,515 | 46.5% (42.1-50.8) | 46.9% (42.6-51.1) | 49.0% (44.3-53.7) | 43.3% (38.8-47.8) |
| IV | 1,970 | 6,515 | 30.7% (25.9-35.5) | 30.4% (25.8-35.1) | 33.7% (28.8-38.6) | 25.1% (20.3-30.0) |
| Missing^1^ | 360 | 6,875 | 5.3% | -- | -- | -- |
| BMI |  |  |  |  |  |  |
| > 18.5 | 2,497 | 5,113 | 49.9% (47.4-52.5) | 49.6% (47.1-52.0) | 46.3% (43.4-49.2) | 54.9% (52.0-57.7) |
| < 18.5 | 2,616 | 5,113 | 50.1% (47.5-52.6) | 50.4% (48.0-52.9) | 53.7% (50.8-56.6) | 45.1% (42.3-48.0) |
| Missing^1^ | 1,762 | 6,875 | 25.6% | -- | -- | -- |
| All | 5,113 | 5,113 | 18.5 (17.1-20.1) | 18.5 (17.1-20.1) | 18.3 (16.9-19.8) | 18.8 (17.3-20.4) |
| CD4 T-cell counts (cells/mm^3^) |  |  |  |  |  |  |
| > 200 | 712 | 4,793 | 14.7% (11.9-17.4) | 15.9% (13.3-18.5) | 14.0% (11.7-16.4) | 19.0% (14.9-23.1) |
| 50 - <200 | 2,206 | 4,793 | 45.7% (42.8-48.7) | 46.9% (44.4-49.4) | 45.9% (43.2-48.6) | 48.7% (45.1-52.2) |
| <50 | 1,875 | 4,793 | 39.6% (36.1-43.1) | 37.2% (33.6-40.7) | 40.1% (36.2-44.0) | 32.3% (28.0-36.6) |
| Missing^1^ | 2,082 | 6,875 | 30.3% | -- | -- | -- |
| All | 4,793 | 4,793 | 72 (27-158) | 78 (30-162) | 69 (27-150) | 96 (35-178) |
| Female | 1,188 | 4,791 | 121 (49-198) | 123 (52-201) | 112 (47-193) | 125 (54-203) |
| Male | 3,603 | 4,791 | 60 (24-137) | 66 (25-143) | 67 (26-146) | 61 (22-133) |
| Hemoglobin (Hgb) |  |  |  |  |  |  |
| > 80 g/L | 4,265 | 4,523 | 93.6% (90.5-96.6) | 94.1% (92.0-96.3) | 93.8% (91.6-96.0) | 94.6% (92.3-97.0) |
| < 80 g/L | 258 | 4,523 | 6.4% (3.4-9.5) | 5.9% (3.7-8.0) | 6.2% (4.0-8.4) | 5.4% (3.0-7.7) |
| Missing^1^ | 2,352 | 6,875 | 34.2% | -- | -- | -- |
| All | 4,523 | 4,523 | 121 (105-136) | 121 (105-136) | 122 (105-138) | 120 (105-134) |
| Hepatitis B (HBV) infection |  |  |  |  |  |  |
| Yes | 454 | 3,340 | 13.9% (12.4-15.5) | 14.2% (11.9-16.4) | 15.5% (12.9-18.1) | 11.9% (9.3-14.5) |
| Missing^1^ | 3,535 | 6,875 | 51.4% | -- | -- | -- |
| Hepatitis C (HCV) infection |  |  |  |  |  |  |
| Yes | 862 | 2,382 | 36.0% (30.9-41.1) | 39.6% (34.8-44.3) | 52.6% (46.7-58.5) | 18.1% (13.5-22.6) |
| Missing^1^ | 4,493 | 6,875 | 65.4% | -- | -- | -- |
| Baseline ART regimen |  |  |  |  |  |  |
| d4T/3TC/NVP | 5,137 | 6,840 | 74.3% (68.9-79.7) | 74.3% (68.9-79.7) | 71.8% (66.1-77.4) | 78.5% (73.0-84.0) |
| d4T/3TC/EFV | 1,157 | 6,840 | 18.6% (14.5-22.7) | 18.6% (14.5-22.7) | 21.7% (17.2-26.2) | 13.6% (9.7-17.5) |
| ZDV/3TC/NVP | 437 | 6,840 | 5.3% (3.6-7.0) | 5.3% (3.6-7.0) | 4.6% (3.0-6.2) | 6.6% (4.3-8.9) |
| ZDV/3TC/EFV | 70 | 6,840 | 1.1% (0.3-1.9) | 1.1% (0.3-1.9) | 1.5% (0.3-2.6) | 0.5% (0.05-0.9) |
| TDF/3TC/EFV/NVP | 21 | 6,840 | 0.4% (0-0.8) | 0.4% (0-0.8) | 0.4% (0-0.7) | 0.4% (0-1.0) |
| Other | 18 | 6,840 | 0.3% (0.1-0.5) | 0.3% (0.1-0.5) | 0.2% (0-0.4) | 0.4% (0-0.9) |
| Unknown | 35 |  | 0.5% | -- | -- | -- |
| Location of facility |  |  |  |  |  |  |
| Urban | 5,769 | 6,875 | 88.1% (80.2-96.1) | 88.1% (80.1-96.1) | 86.5% (77.1-95.9) | 90.8% (84.3-97.3) |
| Rural | 1,106 | 6,875 | 11.9% (3.9-19.8) | 11.9% (3.9-19.9) | 13.5% (4.1-22.9) | 9.2% (2.7-12.7) |
| Facility type |  |  |  |  |  |  |
| Provincial | 3,222 | 6,875 | 41.5% (25.4-57.6) | 41.5% (25.4-57.6) | 38.0% (22.3-53.7) | 47.3% (29.8-64.8) |
| District | 3,653 | 6,875 | 58.5% (42.4-74.6) | 58.5% (42.4-74.6) | 62.0% (46.3-77.7) | 52.7% (35.2-70.2) |
| Site size (number of patients) |  |  |  |  |  |  |
| <200 | 1,160 | 6,875 | 17.2% (8.7-25.8) | 17.2% (8.7-25.8) | 12.1% (5.0-19.3) | 10.1% (2.0-18.3) |
| 200-499 | 3,117 | 6,875 | 38.3% (21.2-55.4) | 38.3% (21.2-55.4) | 32.5% (13.6-51.4) | 34.3% (14.7-53.9) |
| > 500 | 2,598 | 6,875 | 44.4% (29.8-59.1) | 44.4% (29.8-59.1) | 55.4% (37.4-73.4) | 55.6% (36.6-74.6) |
