# Supplementary material for: Outcomes of Antiretroviral Therapy in Vietnam: Results from a National Evaluation
Source: PLoS One. 2013 Feb 15;8(2):e55750. doi: 10.1371/journal.pone.0055750 (PMC3574016; doi:10.1371/journal.pone.0055750)
Supplement: Table S2 — Patient outcomes on ART: retention and immunologic response. Note: These analyses are not based on imputed data. (1) Percentages, 95% confidence interval presented in parentheses. (2) Medians, IQR presented in parentheses. Abbreviation: IDU, intravenous drug use; ART, antiretroviral therapy; CI, confidence interval; IQR, interquartile range; LTFU, loss-to-follow up. (DOCX) [file pone.0055750.s003.docx]

Table S2. Patient outcomes on ART: retention and immunologic response

|  | 6-month | | 12-month | | 24-month | | 36-month | | 48-month | |
| --- | --- | --- | --- | --- | --- | --- | --- | --- | --- | --- |
|  | Median (IQR) or Percentage (95% CI) | *N* | Median (IQR) or Percentage (95% CI) | *N* | Median (IQR) or Percentage (95% CI) | *N* | Median (IQR) or Percentage (95% CI) | *N* | Median (IQR) or Percentage (95% CI) | *N* |
| **Status^1^** |  |  |  |  |  |  |  |  |  |  |
| Alive on ART | 87.5% (85.8-89.0) | 6,785 | 82.6% (80.3-84.7) | 5,511 | 75.9% (72.2-79.2) | 3,218 | 70.2% (64.2-75.6) | 1,371 | 65.2% (53.8-75.2) | 294 |
| Dead | 9.1% (7.7-10.7) | 6,785 | 11.6% (9.8-13.7) | 5,511 | 13.9% (11.5-16.6) | 3,218 | 17.2% (13.6-21.5) | 1,371 | 17.8% (11.5-26.5) | 294 |
| Transfer | 1.0% (0.7-1.4) | 6,785 | 1.7% (1.2-2.4) | 5,511 | 3.7% (2.2-6.2) | 3,218 | 5.9% (3.0-11.4) | 1,371 | 9.8% (3.3-26.0) | 294 |
| LTFU | 2.3% (1.7-2.1) | 6,785 | 4.1% (3.1-5.6) | 5,511 | 6.5% (4.7-9.0) | 3,218 | 6.7% (4.4-10.2) | 1,371 | 7.1% (3.6-15.5) | 294 |
| Stopped treatment | 0.0% | 6,785 | 0.0% | 5,511 | 0.03% (0-0.2) | 3,218 | 0.0% | 1,371 | 0.0% | 294 |
|  |  |  |  |  |  |  |  |  |  |  |
| **Retention^1^** |  |  |  |  |  |  |  |  |  |  |
| All patients | 88.4% (86.8-89.9) | 6,816 | 84.0% (81.8-86.0) | 5,423 | 78.8% (75.7-81.6) | 3,113 | 74.6% (69.6-79.0) | 1,296 | 72.4% (62.9-80.2) | 265 |
| IDU | 86.7% (84.7-88.4) | 2,491 | 81.5% (78.8-84.0) | 2,017 | 73.6% (69.5-77.3) | 1,152 | 67.4% (61.2-73.0) | 521 | 63.3% (49.9-74.9) | 122 |
| Non-IDU | 92.6% (90.2-94.4) | 1,604 | 90.1% (87.2-92.4) | 1,246 | 88.2% (83.3-91.8) | 735 | 88.2% (80.6-93.1) | 317 | 85.7% (74.9-92.3) | 78 |
|  |  |  |  |  |  |  |  |  |  |  |
| **CD4 change^2^** |  |  |  |  |  |  |  |  |  |  |
| All | 94 (45-153) | 2,842 | 142 (78-217) | 2,023 | 213 (120-329) | 1,022 | 254 (135-391) | 371 | 264 (178-372) | 66 |
| Male | 88 (40-142) | 2,083 | 135 (75-205) | 1,475 | 208 (117-321) | 741 | 250 (125-391) | 268 | 261 (164-364) | 55 |
| Female | 114 (60-186) | 759 | 162 (96-263) | 547 | 225 (136-353) | 280 | 260 (162-390) | 103 | 276 (194-579) | 11 |
| Non-IDU | 108 (54-168) | 819 | 152 (87-229) | 573 | 226 (137-369) | 298 | 277 (167-425) | 121 | 238 (195-372) | 27 |
| IDU | 88 (40-142) | 1,147 | 135 (71-200) | 814 | 192 (106-308) | 388 | 228 (110-394) | 146 | 254 (109-368) | 26 |
