# Supplementary material for: Outcomes of Antiretroviral Therapy in Vietnam: Results from a National Evaluation
Source: PLoS One. 2013 Feb 15;8(2):e55750. doi: 10.1371/journal.pone.0055750 (PMC3574016; doi:10.1371/journal.pone.0055750)
Supplement: Table S3 — Factors associated with attrition. (1) average sample size across 20 imputed datasets. Abbreviation: PY, person-years; HR, hazard ratio; AHR, adjusted hazard ratio; IDU, intravenous drug use; ART, antiretroviral therapy; CI, confidence interval; IQR, interquartile range; TB, active tuberculosis; WHO, World Health Organization; BMI, body mass index. (DOCX) [file pone.0055750.s004.docx]

Table S3. Factors associated with attrition

|  | Attrition (All) | | | | | | |  | Attrition (IDU subpopulation) | | | | | | |
| --- | --- | --- | --- | --- | --- | --- | --- | --- | --- | --- | --- | --- | --- | --- | --- |
|  |  |  | Multiple Imputation (*N* = 6,875) | | | | |  |  |  | Multiple Imputation (*N* = 4,273)^1^ | | | | |
|  | N | Rate/ 100PY | Rate/ 100PY | *HR* (95% *CI*) | *p-*value | *AHR* (95% *CI*) | *p-*value |  | N | Rate/ 100PY | Rate/ 100PY | *HR* (95% *CI*) | *p-*value | *AHR*(95% *CI*) | *p-*value |
| **ART start year (per year)** | 6,875 | 12.2 | -- | 0.98 (0.88-1.08) | 0.658 | 0.99 (0.88-1.12) | 0.927 |  | 2,521 | 15.1 | -- | 0.98 (0.89-1.08) | 0.662 | 0.98 (0.88-1.09) | 0.729 |
| **Gender** |  |  |  |  |  |  |  |  |  |  |  |  |  |  |  |
| Female | 1,785 | 5.8 | 5.8 | Reference | -- | Reference | -- |  | 132 | 9.6 | 9.6 | Reference | -- | Reference | -- |
| Male | 5,086 | 14.6 | 14.6 | **2.44 (2.01-2.97)** | **<.001** | **1.59 (1.26-1.99)** | **<.001** |  | 2,388 | 15.4 | 16.2 | **1.64 (1.19-2.25)** | **0.004** | **1.47 (1.08-1.99)** | **0.016** |
| **Age (per year)** | 6,849 | -- | -- | 1.00 (0.98-1.01) | 0.441 | 1.00 (0.99-1.01) | 0.881 |  | -- | -- | -- | 1.00 (0.98-1.02) | 0.962 | 1.00 (0.99-1.02) | 0.661 |
| **IDU** |  |  |  |  |  |  |  |  |  |  |  |  |  |  |  |
| No | 1,618 | 6.5 | 7.0 | Reference | -- | Reference | -- |  | -- | -- | -- | -- | -- | -- | -- |
| Yes | 2,521 | 15.1 | 15.8 | **2.18 (1.75-2.73)** | **<.001** | **1.58 (1.25-2.01)** | **0.002** |  | -- | -- | -- | -- | -- | -- | -- |
| **Active TB** |  |  |  |  |  |  |  |  |  |  |  |  |  |  |  |
| No | 6,055 | 11.6 | 11.6 | Reference | -- | Reference | -- |  | 2,053 | 14.6 | 15.4 | Reference | -- | Reference | -- |
| Yes | 820 | 16.3 | 16.3 | **1.39 (1.18-1.63)** | **0.001** | 1.09 (0.94-1.27) | 0.249 |  | 468 | 16.7 | 17.8 | 1.15 (0.98-1.35) | 0.084 | 1.04 (0.88-1.21) | 0.648 |
| **WHO Stage** |  |  |  |  |  |  |  |  |  |  |  |  |  |  |  |
| Stage I/II | 1,470 | 7.2 | 7.1 | Reference | -- | Reference | -- |  | 433 | 11.7 | 11.3 | Reference | -- | Reference | -- |
| Stage III | 3,075 | 11.7 | 11.8 | **1.67 (1.34-2.08)** | **<.001** | 1.21 (0.99-1.49) | 0.064 |  | 1,144 | 15.1 | 15.0 | **1.34 (1.05-1.70)** | **0.021** | 1.13 (0.89-1.43) | 0.308 |
| Stage IV | 1,970 | 16.7 | 16.8 | **2.44 (1.93-3.09)** | **<.001** | **1.48 (1.22-1.81)** | **<.001** |  | 819 | 16.6 | 19.2 | **1.75 (1.38-2.23)** | **<.001** | **1.29 (1.03-1.62)** | **0.026** |
| **BMI** |  |  |  |  |  |  |  |  |  |  |  |  |  |  |  |
| > 18.5 | 2,497 | 7.2 | 8.0 | Reference | -- | Reference | -- |  | 893 | 10.4 | 11.1 | Reference | -- | Reference | -- |
| < 18.5 | 2,616 | 15.0 | 16.7 | **2.11 (1.81-2.46)** | **<.001** | **1.75 (1.51-2.03)** | **<.001** |  | 980 | 16.0 | 20.0 | **1.84 (1.52-2.22)** | **<.001** | **1.64 (1.38-1.97)** | **<.001** |
| **CD4 T-cell count (cells/mm^3^)** |  |  |  |  |  |  |  |  |  |  |  |  |  |  |  |
| > 200 | 712 | 6.4 | 5.9 | Reference | -- | Reference | -- |  | 237 | 9.7 | 8.2 | Reference | -- | Reference | -- |
| 50 - <200 | 2,206 | 10.8 | 10.2 | **1.74 (1.41-2.14)** | **<.001** | **1.61 (1.28-1.93)** | **<.001** |  | 794 | 12.4 | 13.3 | **1.63 (1.26-2.11)** | **0.001** | **1.56 (1.21-2.01)** | **0.002** |
| <50 | 1,875 | 18.9 | 17.9 | **3.07 (2.44-3.87)** | **<.001** | **2.40 (1.90-3.04)** | **<.001** |  | 807 | 21.4 | 21.7 | **2.64 (2.05-3.38)** | **<.001** | **2.38 (1.86-3.06)** | **<.001** |
| **Hemoglobin** |  |  |  |  |  |  |  |  |  |  |  |  |  |  |  |
| > 80 g/L | 4,265 | 13.5 | 11.7 | Reference | -- | Reference | -- |  | 1,564 | 17.4 | 15.1 | Reference | -- | Reference | -- |
| < 80 g/L | 258 | 25.4 | 22.5 | **1.86 (1.06-3.24)** | **0.028** | 1.47 (0.90-2.41) | 0.120 |  | 110 | 21.8 | 26.8 | **1.71 (1.00-2.91)** | **0.048** | 1.45 (0.93-2.28) | 0.099 |
| **Baseline regimen** |  |  |  |  |  |  |  |  |  |  |  |  |  |  |  |
| Other | 546 | 8.3 | 8.4 | Reference | -- | Reference | -- |  | 199 | 11.1 | 11.5 | Reference | -- | Reference | -- |
| d4T+3TC+NVP/EFV | 6,294 | 12.6 | 12.6 | **1.31 (1.13-1.54)** | **0.001** | 1.17 (0.97-1.40) | 0.092 |  | 2,316 | 15.4 | 16.14 | 1.23 (0.99-1.53) | 0.058 | 1.17 (0.93-1.47) | 0.172 |
| **Location of facility** |  |  |  |  |  |  |  |  |  |  |  |  |  |  |  |
| Urban | 5,769 | 12.1 | 12.1 | Reference | -- | Reference | -- |  | 2,282 | 14.9 | 15.6 | Reference | -- | Reference | -- |
| Rural | 1,106 | 13.3 | 13.3 | 1.12 (0.82-1.52) | 0.469 | 1.07 (0.77-1.49) | 0.667 |  | 239 | 17.9 | 16.9 | 1.11 (0.85-1.44) | 0.435 | 1.14 (0.84-1.54) | 0.401 |
| **Site type** |  |  |  |  |  |  |  |  |  |  |  |  |  |  |  |
| District | 3,653 | 11.6 | 11.6 | Reference | -- | Reference | -- |  | 1,517 | 14.5 | 14.1 | Reference | -- | Reference | -- |
| Provincial | 3,222 | 13.3 | 13.3 | 1.07 (0.79-1.45) | 0.654 | 1.17 (0.89-1.54) | 0.244 |  | 1,004 | 16.4 | 19.0 | 1.22 (0.94-1.60) | 0.134 | 1.23 (0.94-1.62) | 0.128 |
| **Site size** |  |  |  |  |  |  |  |  |  |  |  |  |  |  |  |
| < 200 | 1,160 | 12.7 | 12.7 | Reference | -- | Reference | -- |  | 333 | 18.4 | 15.6 | Reference | -- | Reference | -- |
| 200-499 | 3,117 | 15.5 | 15.5 | 1.17 (0.80-1.70) | 0.401 | 1.20 (0.87-1.66) | 0.246 |  | 964 | 18.7 | 19.8 | 1.21 (0.84-1.73) | 0.294 | 1.20 (0.89-1.62) | 0.225 |
| > 500 | 2,598 | 10.1 | 10.1 | 0.92 (0.63-1.35) | 0.667 | 0.95 (0.63-1.45) | 0.821 |  | 1,224 | 13.1 | 13.4 | 0.98 (0.69-1.40) | 0.926 | 1.02 (0.69-1.52) | 0.914 |
